# Process Development for Hybrid Brake Pedals Using Compression Molding with Integrated In-Mold Assembly

**DOI:** 10.3390/polym17121644

**Published:** 2025-06-13

**Authors:** Deviprasad Chalicheemalapalli Jayasankar, Tim Stallmeister, Julian Lückenkötter, Thomas Tröster, Thorsten Marten

**Affiliations:** Institute for Lightweight Design with Hybrid Systems (ILH), Automotive Lightweight Design (LiA), Paderborn University, Warburger Str. 100, 33098 Paderborn, Germany

**Keywords:** hybrid brake pedals, In-Mold Assembly, compression molding, process development, hybrid structure

## Abstract

Currently, the need for resource efficiency and CO_2_ reduction is growing in industrial production, particularly in the automotive sector. To address this, the industry is focusing on lightweight components that reduce weight without compromising mechanical properties, which are essential for passenger safety. Hybrid designs offer an effective solution by combining weight reduction with improved mechanical performance and functional integration. This study focuses on a one-step manufacturing process that integrates forming and bonding of hybrid systems using compression molding. This approach reduces production time and costs compared to traditional methods. Conventional Post-Mold Assembly (PMA) processes require two separate steps to combine fiber-reinforced plastic (FRP) structures with metal components. In contrast, the novel In-Mold Assembly (IMA) process developed in this study combines forming and bonding in a single step. In the IMA process, glass-mat-reinforced thermoplastic (GMT) is simultaneously formed and bonded between two metal belts during compression molding. The GMT core provides stiffening and load transmission between the metal belts, which handle tensile and compressive stresses. This method allows to produce hybrid structures with optimized material distribution for load-bearing and functional performance. The process was validated by producing a lightweight hybrid brake pedal. Demonstrating its potential for efficient and sustainable automotive production, the developed hybrid brake pedal achieved a 35% weight reduction compared to the steel reference while maintaining mechanical performance under quasi-static loading

## 1. Introduction

The reduction in CO_2_ emissions has become a defining challenge in automotive engineering due to increasingly strong environmental regulations and sustainability targets [1,2,3]. Since vehicle mass directly affects both fuel consumption and emission output, minimizing structural weight has become a core strategy in achieving regulatory and performance goals. This reduction not only contributes to lower greenhouse gas emissions but also enhances vehicle dynamics and extends the electric range in battery-powered vehicle [4,5]. These factors have intensified the development of lightweight materials and innovative hybrid design strategies for use in structural vehicle components [6,7,8]. Composite materials have received considerable attention for their favorable strength-to-weight ratios, with several processing techniques employed to manufacture structural elements [9,10,11,12,13]. Common approaches include hand lay-up, Resin Transfer Molding (RTM), and autoclave curing of prepregs [14,15]. While each has demonstrated value in certain contexts, limitations persist. Hand lay-up is labor-intensive and prone to variation, compromising consistency in structural applications. Prepreg-autoclave processes deliver high mechanical quality but demand costly tooling and extended curing cycles, reducing their suitability for large-scale automotive manufacturing. RTM, though capable of creating moderately complex geometries, relies heavily on precise dry fiber placement and exhibits constrained production rate [16,17,18]. Moreover, the thermosets are inherently brittle, which complicates the integration of functional design features such as localized ribs or interlocking geometries that are critical in load bearing and crash-relevant components. Additionally, once cured, thermosets cannot be reprocessed, which limits recyclability and design flexibility [19]. These drawbacks have driven interest in thermoplastic-based alternatives that offer both toughness and reshaping capabilities under heat [20,21].

Among thermoplastics, glass-mat-reinforced thermoplastics (GMT) present a balanced solution [22,23]. GMT consists of randomly oriented long glass fibers embedded in a thermoplastic matrix, typically polypropylene. When heated, GMT becomes highly formable, enabling the integration of complex features such as rib structures and hollow channels within a single pressing operation [24]. This flowable behavior under compression allows GMT to overcome the brittleness and processing rigidity of thermosets, particularly when used to form geometrically demanding features. Additionally, the absence of a curing stage translates to reduced cycle times and compatibility with high-throughput production lines. GMT’s capability for direct forming under heat and pressure makes it a strong candidate for structural hybrid applications that demand precision, impact resistance, and manufacturability [25,26].

Hybrid material systems that combine metallic substrates with fiber-reinforced thermoplastics (FRP) have emerged as a cost-effective solution for structural applications, especially in crashworthiness [27,28]. Compared to fully composite counterparts, hybrid components deliver a favorable balance among mechanical strength, weight reduction, and manufacturing economy [29]. By strategically applying composite materials only where needed, for instance, as reinforcing ribs or load-transferring elements, material usage is optimized. This targeted reinforcement strategy not only reduces the consumption of expensive composite material but also preserves the structural integrity and high load-bearing capacity afforded by metallic elements. The result is a lightweight yet structurally efficient component that can meet mechanical performance targets without incurring the full cost of all-composite structure.

Despite these advantages, hybrid structures introduce complexity in terms of process integration and post-processing requirements [30]. A significant technical concern arises from the difference in thermal expansion coefficients between metals and polymers, which can induce residual stresses during cooling and affect the durability of the interface [31]. Furthermore, bonding dissimilar materials often necessitates additional joining steps such as adhesive application, mechanical fastening, or thermal welding, each introducing its own processing limitations [32,33]. From a manufacturing perspective, hybrid joining methods can be broadly classified into two categories: post-molding joining (such as mechanical or adhesive bonding) and in situ forming (such as over-molding or co-molding) [34]. The selection of method directly impacts cycle time, labor requirements, and overall system reliability. Compression molding has been widely adopted in the automotive sector for processing thermoplastic composites due to its suitability for forming large, stiff, and lightweight parts. In the context of hybrid structures, one of the most implemented techniques is Post-Mold Assembly (PMA), wherein fiber-reinforced thermoplastic components are formed separately and subsequently bonded to metal substrates. While PMA allows process flexibility and modular tooling, it inherently suffers from prolonged cycle times, alignment challenges, and inconsistent bond quality due to the separate forming and joining stages. These issues become particularly problematic when precise material flow control and tight dimensional tolerances are required. As a result, interest has grown in integrated processes that unify forming and bonding within a single mold cycle, an approach that has the potential to streamline manufacturing, improve interface quality, and reduce production cost.

To address these limitations, a novel manufacturing approach called In-Mold Assembly (IMA) has been developed. In this process, both the forming of the thermoplastic composite and the integration with metallic components occur simultaneously within the compression molding tool, following a principle similar to over-molding [35,36]. In the current study, the effectiveness of this approach was demonstrated through the successful fabrication of a hybrid brake pedal, featuring a GMT-based core structure surrounded by a continuous sheet metal frame. This configuration allowed for efficient integration of the thermoplastic rib geometry with the high-strength metallic load path within a single molding cycle without the need for a secondary joining process.

## 2. Materials and Methods

### 2.1. Materials

The hybrid components developed in this study utilized a thermoplastic matrix composite, stainless steel, and glass-fiber-reinforced polypropylene as the primary constituent materials. Material selection was based on mechanical performance, thermal stability, compatibility for hybridization, and processability within compression molding environments. The composite used for the core structure was a glass-mat-reinforced thermoplastic (GMT) with a polypropylene (PP) matrix. Specifically, the grade S153A248-M1 supplied by Mitsubishi Chemical Advanced Materials (Lenzburg, Switzerland) was used. This material features a nominal glass fiber content of approximately 53 wt.%, composed of long, randomly oriented continuous filaments. The high glass content offers improved tensile and flexural properties, while the PP matrix provides ductility and melt-processability. The semi-finished GMT sheets are suitable for compression molding due to their ability to flow under heat and pressure, allowing complex geometries and localized rib structures to be integrated in a single forming step. Unlike thermoset-based composites, GMT requires no curing, enabling significantly reduced cycle times and reprocess ability of off-cuts, making it a more sustainable and industrially scalable option for automotive lightweight applications. Table 1 Shows the mechanical properties of the GMT used in the current work.

The metal counterpart consisted of austenitic stainless steel 1.4301 (equivalent to AISI 304), delivered with a 2R (bright annealed) surface finish. This steel grade offers good ductility, corrosion resistance, and high formability, making it suitable for integration into the compression molding process. The sheet metal had a nominal thickness of 1 mm, cut to shape via laser cutting. Table 2 shows the mechanical properties of the metal sheet used in the current work. To improve adhesion between the steel and the molten GMT during compression molding, the steel sheets were treated with a thermoplastic-compatible powder bonding system. This coating was applied by Powdertech Surface Science Ltd. (Oxfordshire, UK). The coating facilitates bonding by promoting both mechanical interlock and interfacial compatibility with polypropylene under heat and pressure, eliminating the need for adhesive application or post-mold joining. This combination of materials—PP-based GMT for molded formability and stiffness and 1.4301 stainless steel for load-bearing structural support—offered an effective hybrid solution for producing a lightweight and mechanically robust brake pedal structure.

### 2.2. Demonstrator Geometry

The brake pedal was selected as the demonstrator component for this study due to its safety-critical role in the vehicle structure, its well-defined loading conditions, and its relevance as a mass-produced automotive part requiring both high mechanical performance and weight reduction. Brake pedals must withstand significant bending loads and repeated cyclic loading while maintaining dimensional stability and durability under long-term use. These requirements make the brake pedal an ideal case study for validating a hybrid material system combining a continuous metallic load path with an internally optimized thermoplastic rib structure.

**Figure 1:**
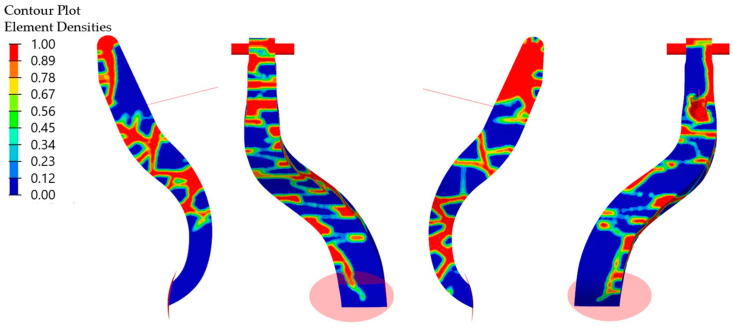
Topology optimization result shows normalized element density from 1.0 (solid) to 0.0 (void), guiding rib placement along critical load paths.

The design of the hybrid brake pedal demonstrator was driven by a topology optimization study aimed at minimizing structural mass while satisfying functional and mechanical performance requirements under defined loading conditions. The optimization process focused on identifying the optimal rib layout and material distribution within the glass-mat-reinforced thermoplastic (GMT) core to ensure sufficient stiffness and load transfer along critical stress paths. Two principal load cases were evaluated: a forward actuation force representing driver-applied pedal press, and a lateral sideload simulating off-axis loading to ensure lateral stiffness and stability. In the simulation model, a concentrated vertical load was applied to the pedal face while a transverse point load introduced lateral bending moments. The rear mounting interface was modeled as a fixed support to replicate installation constraints. Displacement constraints were imposed to limit deflection to 6.44 mm under forward loading and 1.24 mm under lateral loading, ensuring structural integrity during operation. The topology optimization output, illustrated in Figure 1, identified the regions within the pedal core that required reinforcement and those where material could be eliminated without compromising stiffness. The optimization was based on a density method, in which the element density represents a unitless, normalized variable ranging from 1.0 (fully solid material) to values approaching 0.0 (void regions where material is removed). This density map provided a quantitative basis for defining the rib structure geometry along the principal load paths. Based on the optimization results, a uniform rib thickness of 4 mm was selected as a design compromise that balanced mechanical stiffness, mass efficiency, and manufacturability. The thickness was determined as the minimum viable dimension capable of satisfying the displacement constraints (6.44 mm push, 1.24 mm sideload) while minimizing material use and ensuring processability. This dimension also ensured reliable mold filling during compression molding, preventing defects such as incomplete rib formation or voids. The rib layout and thickness were directly transferred into the mold cavity design, allowing replication of the optimized structure in a single-step compression molding operation.

**Figure 2:**
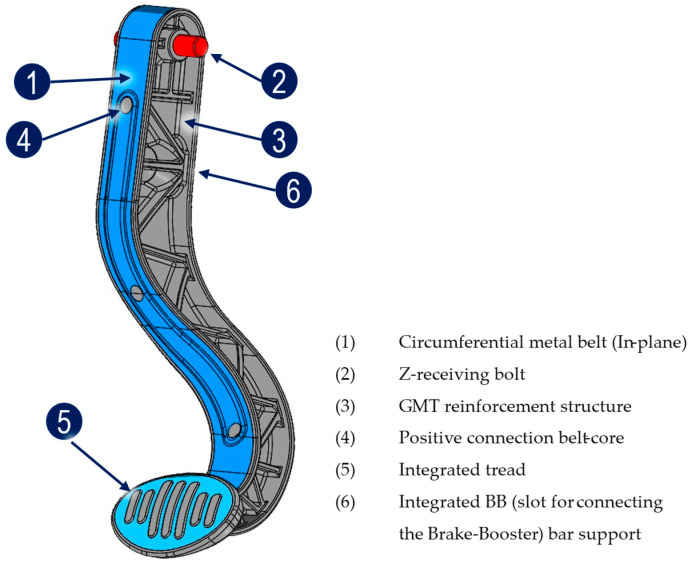
Hybrid brake pedal demonstrator highlighting key structural and functional features.

The resulting pedal geometry integrated the optimized rib structure into a functional, manufacturable hybrid design. As shown in Figure 2, the demonstrator incorporates seven key functional zones. A continuous outer steel belt forms the load-bearing boundary, carrying tensile and compressive stresses under bending loads. Inside this metallic frame, the optimized ribbed GMT core enhances bending stiffness and distributes shear stresses. To achieve mechanical interlocking between the steel and thermoplastic layers, perforations were incorporated into the steel belt, allowing the molten GMT to flow through and mechanically anchor during compression molding. The pedal face region was locally thickened in the thermoplastic layer to withstand localized input forces during driver actuation. Integrated mounting interfaces allow direct attachment to link components without requiring secondary joining operations. A critical feature of the geometry is the snap-fit connection zone designed for irreversible coupling with the brake booster pin; this undercut geometry, oriented perpendicular to the press direction, required dedicated mold mechanisms for transverse demolding. Through the combination of topology-driven rib design, integrated functional features, and hybrid material architecture, the brake pedal demonstrator exemplifies a structurally optimized, lightweight component suitable for safety-critical automotive applications.

### 2.3. Tool and Mold Design

**Figure 3:**
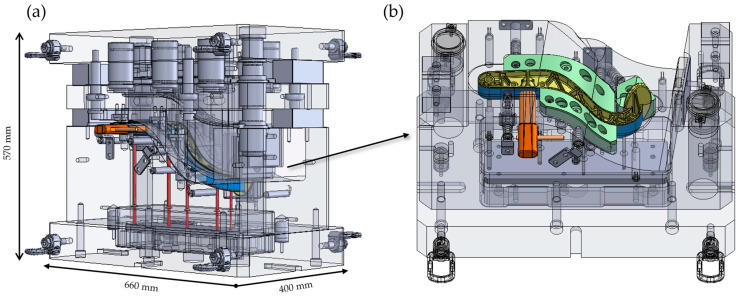
Tooling used for producing the hybrid brake pedal. (**a**) Full view of the compression molding tool with the brake pedal positioned inside the mold cavity, including the location of the integrated brake booster modular section and pin interface. (**b**) Detailed view showing the lower mold cavity and the positioning of the modular brake booster section within the tool. The green components represent movable inserts that compress during tool closure and expand upon tool opening, assisted by integrated springs, facilitating part ejection after molding.

**Figure 4:**
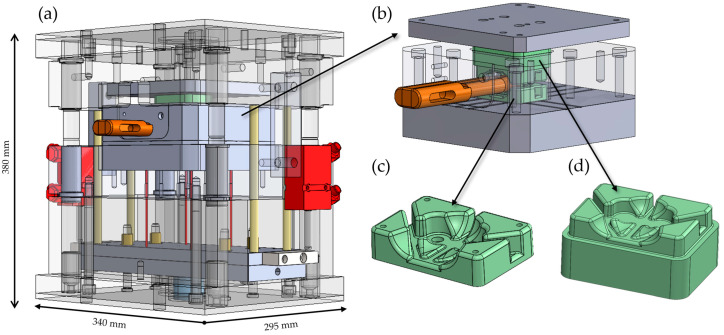
Tooling used for manufacturing the modular brake booster pin connection section. (**a**) Full view of the dedicated tool, showing the cavity location (grey), cavity insert (orange), and hydraulic ejection system components (red). (**b**) Closer view of the modular insert’s integration position within the mold setup. (**c**) Lower section of the modular insert cavity. (**d**) Upper tool section of the modular insert.

The mold system for producing the hybrid brake pedal demonstrator was developed to address the complexity of integrating a metallic belt and a thermoplastic rib structure within a single compression molding process. The tooling strategy incorporated modularity, functional flexibility, and adaptability for iterative testing. In total, three distinct compression molding tools were designed and fabricated to support different stages of process development and validation. The primary tool full-scale mold for the hybrid brake pedal was engineered and constructed by Scheideler GmbH & Co. KG (Borgentreich, Germany) to replicate the complex rib geometry and integrate the continuous steel belt with the GMT core in a single molding cycle. A key design feature of this mold was the inclusion of a modular insert in the brake booster connection area, allowing interchangeable cavity configurations for that critical interface. This modularity enabled the brake booster slot geometry to be modified or replaced independently of the main tool cavity, providing flexibility to iterate the design or correct functional issues without requiring complete mold rework as shown in Figure 3. Furthermore, the modular insert could be detached from the main mold assembly and operated as a standalone mold cavity, enabling independent production of the booster connection region for experimental testing and validation of the snap-fit geometry and connection performance prior to integration into the full pedal mold. A second dedicated mold was fabricated using this modular insert as a standalone compression molding tool to support early-stage process validation of the brake booster interface as shown in Figure 4. This configuration allowed dimensional analysis and functional testing of the snap-fit feature under simplified molding conditions before committing to full-scale part production.

A third compression molding tool was developed by the University of Paderborn for the fabrication of flat hybrid laminate samples to investigate the interface bonding behavior between the steel substrate and GMT. This flat-panel mold featured a fixed cavity depth and integrated heating to produce standardized laminate specimens for adhesion testing, such as shear and peel strength characterization. These flat samples enabled systematic evaluation of the adhesion-promoting powder coating applied to the steel surface, independently of the geometric complexity of the brake pedal.

All three molds were equipped with a hydraulic ejection system to facilitate demolding of the formed components. Ejector pins were integrated into each tool and controlled via hydraulic actuation to ensure reliable, uniform part ejection without damaging delicate rib structures or interlocking geometries. The main pedal mold incorporated precision alignment features and mechanical stops to secure the steel insert during molding, preventing displacement under compression. Electric heating elements were embedded within all molds to maintain isothermal processing conditions, monitored via thermocouples distributed across the mold cavity. The combined modular and multi-tool approach to mold design enabled iterative validation of critical connection interfaces, adhesive bonding performance, and final part geometry within a scalable and adaptable compression molding framework.

### 2.4. Sample Fabrication and Processing

In the current work, three different types of samples were produced to address distinct stages of process validation and functional evaluation. Each sample type was manufactured using a dedicated compression molding tool tailored for its specific geometry and testing purpose. The first type consisted of flat hybrid laminate specimens, prepared to investigate the interfacial bonding behavior between glass-mat-reinforced thermoplastic (GMT) and the powder-coated steel substrate under controlled planar conditions. The second sample type comprised brake booster pin connection specimens, molded independently to validate the dimensional accuracy and mechanical performance of the snap-fit connection prior to full integration into the brake pedal assembly. The third and final fabrication stage involved the production of the full hybrid brake pedal demonstrator, incorporating the steel belt, GMT rib structure, and integrated booster connection zone into a single molded component. Each sample type followed a tailored fabrication process, including material preheating, controlled compression molding, and post-mold handling. The following sections describe the specific fabrication steps, processing parameters, and tooling configurations used for each sample type.

#### 2.4.1. Interface Bond Testing Samples

**Figure 5:**
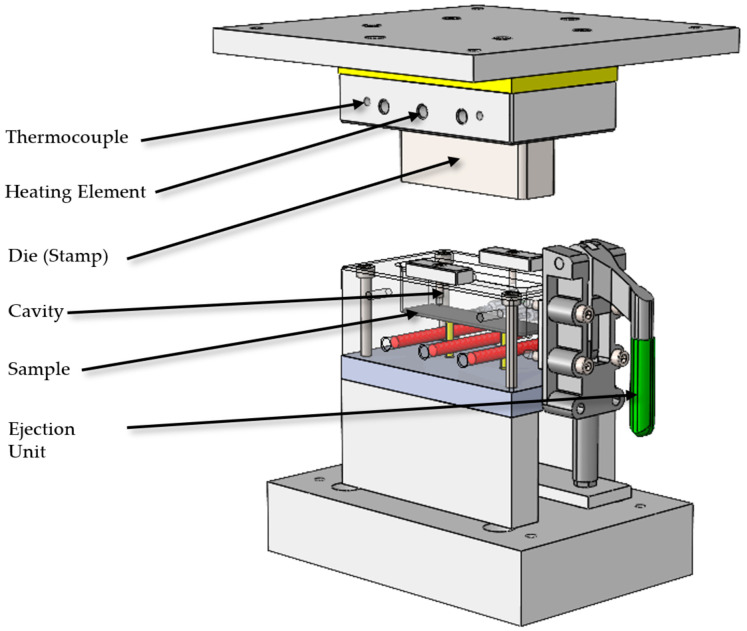
Compression molding tool used for producing flat hybrid specimens for interface bond testing.

To produce flat hybrid specimens for interfacial bond evaluation between glass-mat-reinforced thermoplastic (GMT) and powder-coated stainless steel, a dedicated compression molding tool was developed and utilized at the University of Paderborn. An image of this tool is shown in Figure 5. The cavity surface area of the tool measured 145 mm × 40 mm, providing simplified and material-efficient geometry for producing bonded laminate samples under well-controlled process conditions. Stainless steel blanks, coated with a polypropylene-compatible adhesion promoter, were supplied by Powdertech Surface Science Ltd. (Oxfordshire, UK). These sheets were thermally activated using an infrared radiation system, which heated the coated surface for 60 s to a target temperature of approximately 180 °C. Simultaneously, GMT sheets were preheated in a convection oven at 250 °C for 10 min to reach a sufficiently softened state for molding.

Immediately after heating, both materials were placed in the mold cavity. The tool was then closed, and a specific pressure of 150 bar (86 kN) was applied and held for 60 s under isothermal compression molding conditions. The tool was electrically heated to maintain the required process temperature of 100 °C throughout the cycle. Upon completion of the molding cycle, the hybrid sample was manually demolded using an integrated mechanical ejection system, where ejector pins were actuated via a lever mechanism to safely push the sample out of the cavity. This setup enabled controlled and damage-free part removal. The resulting flat hybrid panels were produced with high repeatability and consistency, providing a reliable basis for the adhesion performance evaluation, which will be discussed in the following chapter.

#### 2.4.2. Brake Booster Pin Connection Samples

To validate the geometric feasibility and molding conditions of the brake booster pin interface, particularly the snap-fit connection zone, dedicated sample production was carried out using a specialized tool insert developed for this purpose. The tool was designed collaboratively by EMS GmbH & Co. KG (Borgentreich, Germany) and the University of Paderborn. Scheideler was responsible for designing and fabricating the brake booster cavity and undercut mechanism, while the remaining mold components were developed by the university. This setup allowed the production of isolated snap-fit specimens prior to their integration into the full hybrid brake pedal mold. The booster pin region presents a significant geometric challenge because the snap-fit feature must be molded orthogonally to the main press direction. Additionally, the thermoplastic must form around a transverse undercut, which would normally obstruct part ejection. To address this, a modular tool insert was developed containing a three-piece sliding core system. During demolding, the center core is manually withdrawn first, followed by the removal of the two outer sliding elements. This sequence enables safe release of the undercut geometry from the cavity. The entire slider mechanism is actuated by hand and mechanically locked in place to withstand the compression forces applied during molding. In industrial production scenarios, this system could be adapted for hydraulic actuation and synchronized with the press cycle.

**Figure 6:**
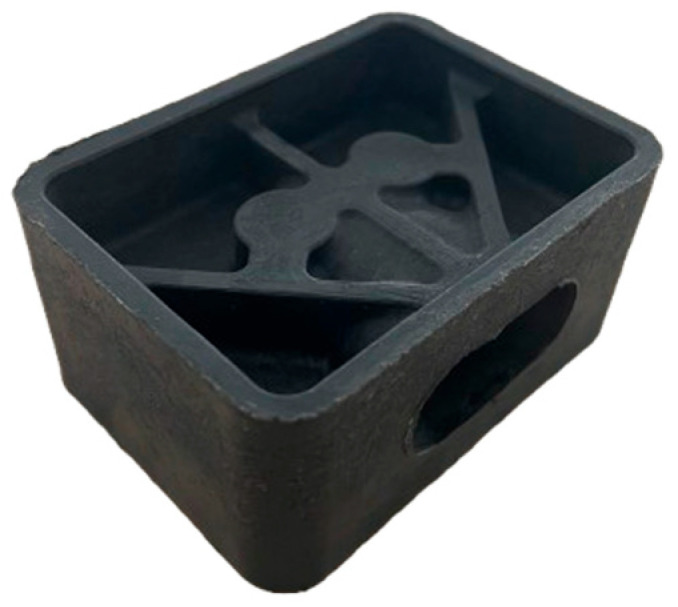
Section of the brake pedal geometry produced using a modular mold tool to validate formability of undercut features.

During the sample production process, GMT sheets were preheated for 10 min at 250 °C in a convection oven. Once softened, the GMT was placed into the mold cavity. The forming operation was conducted at a molding pressure of 100 bar and maintained for 60 s under isothermal conditions. The tool was electrically heated to ensure consistent cavity temperature throughout the process. After solidification, the molded parts were manually ejected using a mechanical ejection system as shown in Figure 6. The sliding core was disengaged by hand and removed without resistance, confirming smooth demolding at the selected pressure level. This dedicated tooling configuration allowed for early validation of the snap-fit undercut geometry and mold filling performance in the brake booster interface area. Isolating this critical section minimized material consumption and enabled design refinement before incorporating the insert into the complete hybrid brake pedal mold.

#### 2.4.3. Hybrid Brake Pedal Demonstrator

Following completion of the thermal studies and tool setup, the full-scale compression molding tool was prepared for manufacturing the hybrid brake pedal demonstrators. The tool was equipped with a hydraulic ejector system to enable complete demolding of the hybrid structure via an integrated ejector unit. The required mass of GMT was approximated using a CAD-based volume estimation and totaled approximately 317 g. Based on the cavity geometry, a preform contour was generated to optimize material placement within the mold. This contour was waterjet-cut from flat GMT sheets to achieve a precise fit. Each hybrid brake pedal required five full layers of GMT, each weighing approximately 60 g. In the region near the pedal face, where the cavity depth increases significantly, an additional two localized reinforcing patches were added to ensure sufficient filling. These smaller pieces compensated for material accumulation challenges in the deep draw area, bringing the total inserted GMT mass to 320 g.

**Figure 7:**
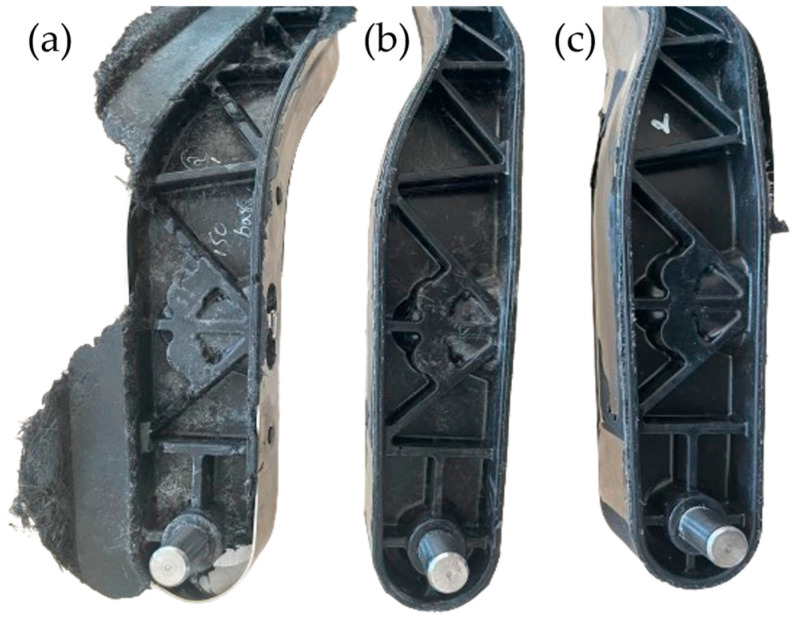
Effect of processing parameters on hybrid brake pedal quality. (**a**) Incomplete filling due to insufficient material input and pressure. (**b**) Material degradation observed from poor thermal control with increased pressure and material. (**c**) Improved filling and surface quality achieved with increased tool temperature.

Initial molding trials were conducted using a GMT stack of five full layers and two partial layers, resulting in a total material mass below the required threshold. These trials were carried out at a compression pressure of 150 bar and a tool temperature of 100 °C. Under these conditions, incomplete filling was observed in the rib and core zones due to insufficient material and pressure (see Figure 7a). In the second trial, the same layer configuration was retained, but the compression pressure was increased to 250 bar. This resolved the core filling issue and achieved complete cavity saturation. However, the higher pressure led to localized fiber accumulation and material degradation near undercuts and the pedal zone (see Figure 7b). To address these issues, the tool temperature was increased to 120 °C in the third trial, while keeping the pressure and material stack constant. This adjustment significantly improved material flow, enabling full rib replication without localized fiber build-up (see Figure 7c). Throughout all trials, tool displacement over time was monitored. The mold closure followed a consistent linear displacement curve with minimal deviation, confirming stable tool operation and proper shrinkage compensation.

**Figure 8:**
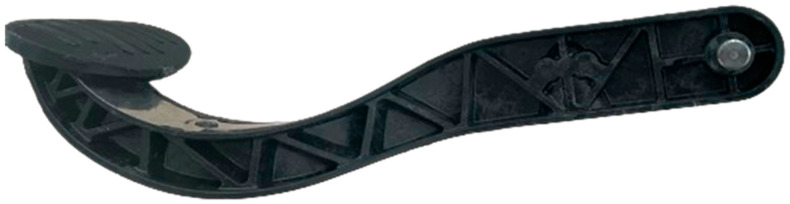
The hybrid brake pedal shown after post-processing. Excess flash from compression molding was removed, and the final geometry was achieved using the Ultrasonic 65 monoBLOCK machine from DMG MORI GmbH (Bielefeld, Germany).

Despite successful process control in the core region, the pedal face area presented persistent molding challenges. The cavity in this zone requires significant material flow in the negative z-direction, which proved difficult to achieve consistently, even with localized GMT patching. Preforming additional layers was insufficient to completely fill the deep geometry of the pedal section. This limitation is attributed to complex cavity geometry and restricted vertical material movement, as visualized in the flow analysis. To overcome this issue, the pedal face section was manufactured separately using Fused Deposition Modeling (FDM). Fused Deposition Modeling (FDM) is an additive manufacturing process in which thermoplastic filament is extruded layer by layer to form 3D objects. It is widely used for producing functional prototypes and structural parts due to its speed and material flexibility. In this study, a polyamide (PA) part was fabricated via FDM to address incomplete material flow in the pedal face region. The PA filament, supplied by Bambu Lab, provided the necessary toughness, thermal stability, and dimensional accuracy for integration into the hybrid structure. The printed part was designed with strategic openings and recesses to allow molten GMT to flow around and through the structure during compression molding, resulting in a mechanically interlocked interface between the ribbed core and the additively manufactured surface. Printing was carried out at 230 °C with a 70 °C bed temperature, using a 0.2 mm layer height and horizontal build orientation to promote optimal bonding during molding. This printed part was placed into the mold prior to compression and successfully integrated into the final hybrid component. This approach ensured complete filling and functional integrity of the pedal area, while maintaining process compatibility within the compression molding workflow. Table 3 summarizes selected key molding trials conducted in the current study. Trial 5 represents the optimized parameter set used for final production. Two pedal types were ultimately produced using this hybrid process approach: one relying solely on mechanical interlock between the steel belt and GMT, and a second incorporating both mechanical and material bonding. The integration of functional features and material-specific strategies enabled successful demonstration of a structurally optimized hybrid brake pedal geometry using a combined compression molding and additive manufacturing route. Figure 8 shows the produced hybrid brake pedal after post-processing, where excess material was trimmed using an Ultrasonic 65 monoBLOCK machine from DMG MORI GmbH (Bielefeld, Germany).

## 3. Results

### 3.1. Interface Bond Results

**Figure 9:**
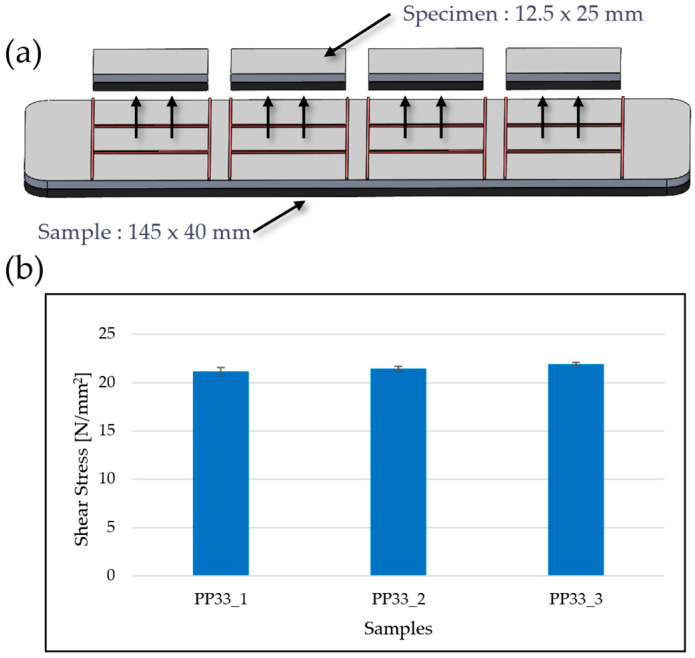
Schematic of the interface bond test setup (**a**) and corresponding shear strength results (**b**) for three sample sets. Each specimen measures 12.5 × 25 mm, bonded to a 145 × 40 mm base sample.

The interface bonding strength between the GMT and powder-coated stainless steel was evaluated through compression-shear testing of flat hybrid specimens. The rectangular test specimens, measuring 12.5 mm × 25 mm, were extracted from larger molded panels using a wet abrasive cut-off saw (Figure 9a). Testing was conducted using a compression-shear setup, applying load perpendicular to the bonding interface to quantify the interfacial strength of the adhesion promoter. The results demonstrated excellent adhesion between the GMT and steel. Maximum shear strengths reached up to 22 MPa, with failure typically occurring cohesively within the GMT rather than at the interface, as shown in Figure 9b. This indicates strong chemical compatibility and sufficient thermal activation of the bonding system.

### 3.2. Brake Booster Pin Connection Results

**Figure 10:**
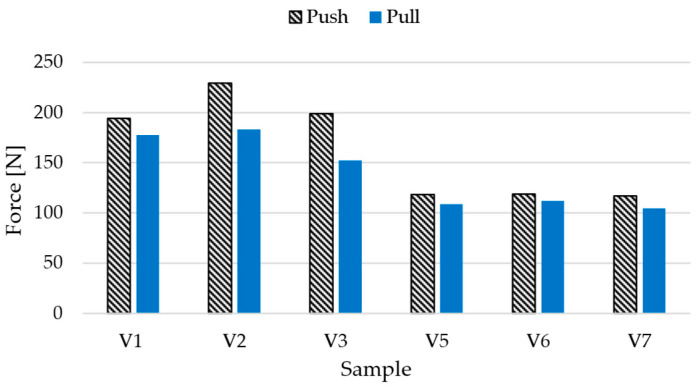
Comparison of push and pull forces for two different joint diameters.

To evaluate the mechanical performance of the brake booster pin connection, insertion (push) and removal (pull) forces were measured for pins of two different oversize configurations relative to a fixed cavity diameter of 16.00 mm. Specifically, pins with diameters of 16.32 mm (2% oversize, Samples V1–V3) and 16.16 mm (1% oversize, Samples V5–V7) were tested. The goal was to ensure a secure snap-fit connection between the brake booster rod and the pedal bracket while maintaining ease of assembly. As shown in Figure 10, insertion forces remained below 230 N across all tested samples, with the highest values recorded for the 2% oversized group. This confirms that the connection can be installed without the need for specialized mechanical tools, fulfilling the requirement for simple manual assembly. The chosen ball-head design of the pin also eliminates the need for auxiliary fastening components such as washers or centering pins, contributing to assembly simplification. To further validate the robustness of the joint, a three-cycle fatigue test was conducted on representative samples. No measurable degradation in pull-out force was observed, indicating that the connection mechanism retains its structural integrity under repeated load cycles and does not exhibit wear or damage after multiple insertions and removals. While the current evaluation focused on short-term assembly–disassembly behavior, the long-term fatigue performance of the snap-fit connection was not assessed. This aspect is critical for safety-relevant applications and will be addressed in future studies.

### 3.3. Thermal Analysis of the Hybrid Tool

**Figure 11:**
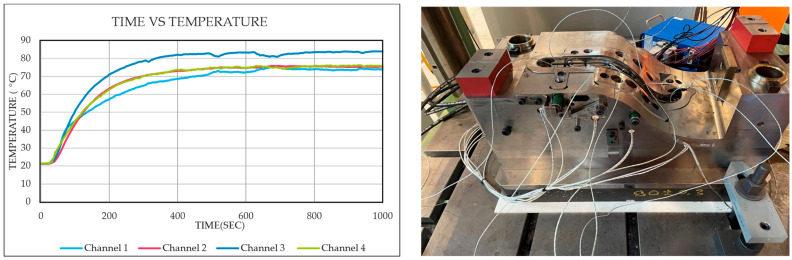
Time–temperature response (**left**) and thermocouple-instrumented mold setup (**right**) used for monitoring thermal distribution during compression molding.

A thermal analysis was conducted to assess heat transfer from the electrically heated mold to the stainless-steel insert during the molding process. To record surface temperature changes, four thermocouples were spot-welded directly onto different locations of the steel belt insert, which was placed into the lower mold half. With the tool held at a nominal temperature of 100 °C, the measured temperatures on the steel insert ranged from 73 °C to 82 °C, depending on the measurement point. This variation in heat transfer is attributed to two main factors. First, heat loss occurs at the open surface of the mold cavity due to exposure to ambient air. Second, contact between the spring back-curved metal belt and the tool surface is not uniform across the full contact area, which results in location-specific heat conduction differences. The local deviation from the tool’s internal setpoint temperature (100 °C) illustrates the impact of surface contact conditions on the thermal activation of the interface, as shown in Figure 11. To compensate for this discrepancy, the tool temperature was increased to 110 °C to achieve approximately 100 °C on the steel insert surface and further raised to 135 °C to reach 120 °C on the insert during the production process. Prior to molding, the tool was preheated for approximately 90 min to ensure thermal stabilization and uniform heat distribution across all surfaces.

### 3.4. Hybrid Brake Pedal Fabrication Results

**Figure 12:**
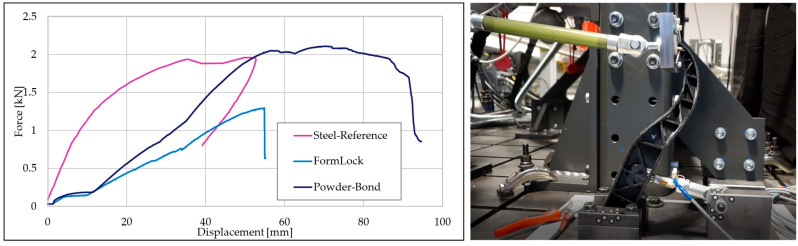
Force–displacement curves (**left**) comparing steel, form-lock hybrid, and powder-bonded hybrid brake pedals. The (**right**) image shows the reversed test-bench setup used for quasi-static compression testing, where forces were measured at the coupling rod.

**Figure 13:**
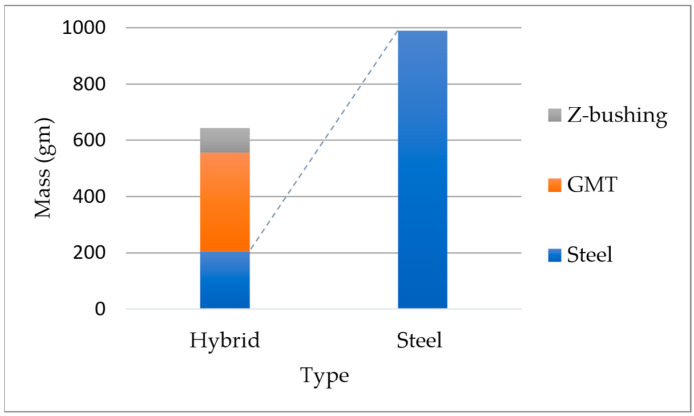
Mass comparison between the hybrid and steel brake pedal designs, showing component-wise contribution (steel, GMT, and Z-bushing).

This section presents the test results from the hybrid brake pedal demonstrators, focusing on force–displacement behavior, visual failure analysis, and overall component validation. Two hybrid variants were investigated: one based on form-fit mechanical interlocking between GMT and steel, and another combining form-fit and chemical bonding using a PP-based adhesion promoter. Both pedal variants were subjected to quasi-static compression tests using a custom-built test rig (see Figure 12). In this setup, the pedals were tested in a reversed orientation to replicate the inverse load path of an actual vehicle brake assembly. Force was applied at the pedal pad via a linear actuator, while the reaction force was measured at the coupling rod using a calibrated force sensor. This arrangement mirrors the force transmission through the pushrod to the brake booster in real-world conditions. The pedals were fixed in position, and force was applied to the pedal face at a rate of 10 mm/min. In both hybrid configurations, delamination was observed between the GMT and the metallic belt in the region near the brake booster pin interface. For the form-fit-only pedal, detachment occurred at a displacement of 55 mm, with a recorded maximum force of 1.32 kN, corresponding to 37% lower strength than the steel benchmark. In contrast, the powder-bond-enhanced pedal demonstrated improved integrity, achieving a maximum force of 2.15 kN at 64 mm displacement, slightly exceeding the force capacity of the steel pedal by 2%. All tests were performed in triplicate for each pedal type, with consistent results observed within a 1% tolerance range, confirming the repeatability and reliability of the findings. From a mass perspective, the hybrid brake pedal weighs 642 g, which represents a 35% weight reduction compared to the steel reference pedal (990 g). The component’s material composition is illustrated in Figure 13, with GMT forming the ribbed core, a steel belt for structural support. This hybrid construction meets the current work’s lightweighting objectives without compromising structural performance under quasi-static loading. The chosen demonstrator geometry proved to be highly representative of automotive hybrid component challenges. It integrates mechanical function, geometric complexity, and hybrid joining in a single molding process.

## 4. Discussion

The findings of this study confirm the practical feasibility and highlight certain limitations of compression-molded hybrid structures for automotive applications, specifically in brake pedal manufacturing, and reflect broader insights gained from other evaluated components in this project. The hybrid design effectively combines metallic and thermoplastic components through In-Mold Assembly (IMA). Each evaluated development area contributed to validating the component concept and the associated manufacturing methodology. The interface bonding results confirmed that a PP-compatible powder bond system can reliably produce strong adhesion between stainless steel and GMT. Achieving bond strengths up to 22 MPa and observing cohesive failure in the GMT matrix reflect a mechanically robust interface that was properly activated during processing. These findings support the working hypothesis that coupling mechanical interlocking with chemical adhesion can significantly enhance joint integrity, aligning with prior knowledge on multi-material interfaces in thermoplastic hybrid systems. In the booster pin section, the modular slider-based tool successfully demonstrated the feasibility of forming undercut geometries perpendicular to the press direction, thereby validating the broader concept of hybrid integration using sliding-core tooling. This approach offers a distinct advantage over conventional multi-stage joining methods.

The thermal analysis uncovered non-uniform heating at the steel insert surface, with temperatures ranging between 73 and 82 °C despite a tool setpoint of 100 °C. This discrepancy is attributed to insufficient thermal contact and environmental heat loss, underscoring the need for optimized thermal management strategies. Future designs should explore localized heating methods, improved fixture, or elevated base tool temperatures to ensure uniform temperature profiles and consistent interface activation. The final hybrid brake pedal demonstrators reflected both the potential and challenges of the integrated molding process. While the rib structure was successfully molded at 250 bar and 120 °C, incomplete material flow into the pedal cavity led to partial filling. The introduction of a 3D-printed polyamide inserts mitigated structural limitations by providing a mechanical connection, enabling proper formation of the critical load-bearing zone. The powder-bonded pedal variant reached a peak force of 2.15 kN, exceeding the benchmark steel pedal, whereas the form-fit-only version reached 1.32 kN, confirming the performance benefit of enhanced adhesion at the interface. Additionally, the 35% mass reduction highlights the lightweighting potential of GMT while maintaining mechanical integrity, reinforcing the value of hybrid solutions in next-generation vehicle design. Beyond proving technical feasibility, this study demonstrates promising industrial implications. The hybrid approach eliminates multiple joining steps and reduces part count compared to conventional welded steel assemblies, which could lead to substantial reductions in energy consumption and tooling complexity in high-volume production.

While the feasibility of compression molding was demonstrated, a detailed investigation of production parameters remains necessary. Although compression pressure was evaluated in this study, other critical variables such as tool temperature and the influence of different thermoplastic types within the GMT were not explored in depth. Future work should address these factors to better understand their impact on part quality and process reliability. Additionally, the current tool must be redesigned to enable complete pedal face formation using GMT alone, for higher functional integration through one-shot molding. In summary, while the hybrid structure presented here marks a significant step toward lightweight and functionally integrated components, further refinement in tool design and parameter study is essential. These efforts will support the broader applicability of this concept to other automotive components such as seat structures, floor modules, and suspension systems, thereby advancing industry goals in sustainable lightweight design. This study provides a practical pathway for integrating lightweight thermoplastic composites with metallic load paths in a single molding step, which is highly relevant for researchers exploring efficient hybrid manufacturing. The presented approach can serve as a foundation for further development in safety-critical automotive applications and scalable lightweight design strategies.

## Figures and Tables

**Table 1:** Material properties of glass-mat-reinforced thermoplastic (S153A248-M1) ^1^.

Tensile Strength	Elongation Rate at Break	Flexural Modulus	Density (Molded)
145 MPa	2.3	8400 MPa	1.4 g/cm^3^

^1^ All material property values presented are based on technical datasheets provided by the respective manufacturers.

**Table 2:** Mechanical properties of austenitic stainless steel 1.4301 (2R finish) ^1^.

Tensile Strength	Elongation Rate at Break	Young’s Modulus	Density (Molded)	Thickness
500–700 MPa	35 ± 5%	200 GPa	7.9 g/cm^3^	1 mm

^1^ All material property values presented are based on technical datasheets provided by the respective manufacturers.

**Table 3:** Summary of compression molding trials and outcomes.

Trial No.	GMT Layers Used	Tool Temperature (°C)	Pressure (bar)	Material Input (g)	Outcome Description
1	5 full	100	150	300	Incomplete filling in rib and core regions due to insufficient material input.
2	5 full + 2 patches	100	150	320	Improved material distribution, but rib and core zones still exhibited underfilling.
3	5 full + 2 patches	100	250	320	Rib and core fully filled; however, fiber accumulation and degradation were observed. Pedal zone remained incomplete.
4	5 full + 2 patches	120	250	320	Enhanced material flow and surface quality; full rib replication, but pedal zone still underfilled.
5	5 full	120	250	300	Complete filling of all regions; final hybrid brake pedal geometry successfully produced.

## Data Availability

The original contributions presented in the study are included in the article; further inquiries can be directed to the corresponding author.

## Abbreviations

The following abbreviations are used in this manuscript:

GMT	Glass Mat Thermoplastic
FDM	Fused Deposition Modeling
RTM	Resin Transfer Molding
IMA	In-Mold Assembly
PA	Polyamide
PP	Polypropylene
